# Segmental Bioimpedance Phase Angles for Frailty Detection in Hospitalized Older Adults with Cardiovascular Disease: A Cross-Sectional Observational Study

**DOI:** 10.3390/healthcare13212816

**Published:** 2025-11-06

**Authors:** Noel Rivas-González, Mª José Castro, Irene Albertos, María López, Belén Martín-Gil, Elsa Rodríguez-Gabella, Mercedes Fernández-Castro, J. Alberto San Román

**Affiliations:** 1 Continuing Education Department, Valladolid University Clinical Hospital, 47005 Valladolid, Spain; nrivas@saludcastillayleon.es; 2 Valladolid Biosanitary Research Institute (IBIOVALL), Valladolid University Clinical Hospital, Calle Rondilla Santa Teresa, 47010 Valladolid, Spain; bmartingi@saludcastillayleon.es (B.M.-G.); mefernandezc@saludcastillayleon.es (M.F.-C.); asanroman@secardiologia.es (J.A.S.R.); 3 Faculty of Nursing, University of Valladolid, 47003 Valladolid, Spain; irene.albertos@uva.es (I.A.); maria.lopez.vallecillo@uva.es (M.L.); 4 GIR Research Group on Multidisciplinary Assessment and Intervention in Health Care and Sustainable Lifestyles, University of Valladolid, 47003 Valladolid, Spain; 5 Department of Nursing Care Information Systems, Valladolid University Clinical Hospital, 47005 Valladolid, Spain; erodriguezga@saludcastillayleon.es; 6 Research Support Unit, Valladolid University Clinical Hospital, 47005 Valladolid, Spain; 7 Cardiology Department, Valladolid University Clinical Hospital, 47005 Valladolid, Spain; 8 Centro de Investigación en Red de Enfermedades Cardiovasculares (CIBERCV), 28029 Madrid, Spain

**Keywords:** phase angle, bioimpedance, body composition, cardiovascular disease, older adult, hospitalization

## Abstract

**Highlights:**

**What are the main findings?**

- Segmental bioimpedance phase angles are significantly lower in frail older adults hospitalized with cardiovascular disease.
- In men, left body and left leg phase angles showed moderate discriminatory capacity for frailty detection, while women showed lower predictive performance.

**What are the implications of the main findings?**

- Segmental phase angle measurement is a simple, rapid, and non-invasive bedside tool that can support early frailty identification in cardiology wards.
- Integrating phase angles with routine biomarkers such as hemoglobin and CRP could improve patient risk stratification, optimize resource allocation, and guide individualized care strategies in hospital settings.

**Abstract:**

**Background/Objectives**: Whole-body phase angle is associated with in-hospital morbidity and mortality, although cut-off points vary. Studies on the relationship between segmental phase angles and frailty in patients with cardiovascular disease are limited. Therefore, we aimed to assess the prognostic value of segmental phase angles in detecting frailty in older adults hospitalized with cardiovascular disease. **Methods**: A cross-sectional observational study was conducted on hospitalized patients aged ≥60 years with cardiovascular disease (sample size: 117–158 subjects). Frailty was identified using Fried’s five criteria. Biomarkers, body composition, and segmental phase angles were assessed using multifrequency bioimpedance. Associations with frailty were analyzed using logistic regression and Receiver Operating Characteristic (ROC) curves. Sensitivity, specificity, and positive likelihood ratio (LR+) were calculated (95% CI; *p* < 0.05). **Results**: A total of 157 patients (men: 64.24%; women: 33.76%) were included, with a mean age of 73.23 years (SD = 7.91). The prevalence of frailty was 28.66%. In men, the phase angles of the left hemisphere (5.15°) and left leg (4.25°) demonstrated moderate-accuracy capacity (AUC: 0.66–0.71; LR+: >2). In women, the segments with significance did not exceed an LR+ of 2. Frailty was associated with lower phase angle values in all segments and biomarkers such as hemoglobin < 12 g/dL (*p* = 0.011) and CRP > 5 mg/L (*p* = 0.030). **Conclusions**: Segmental phase angles demonstrated moderate discriminatory capacity for identifying frailty among hospitalized older men with cardiovascular disease, though predictive capacity in women was limited. This approach may support bedside frailty screening and inform individualized management.

## 1. Introduction

Clinical nutrition is the result of the interaction between food deprivation and the catabolic processes that occur during illness and aging; of special importance are those processes in which nutrition plays a significant role, such as in the care of patients with cardiovascular disease (CVD), diabetes mellitus, or dyslipidemia, among others [1].

In general, the impact of age from 65 years onward influences body composition, affecting lean mass, as well as water and bone mineral mass [2], although it is currently estimated that these changes begin at 60 years, with a significant decrease in muscle mass index and strength, with a consequent increase in the risk of sarcopenia. In turn, sarcopenic obesity is associated with an increase in cardiovascular risk, among other consequences [3].

Frailty has been directly linked to aging and is a functional risk factor for cardiovascular morbidity and mortality [4]. Several studies have sought to explore the relationship between nutrition and frailty through the biological processes involved, such as oxidative stress and inflammation, among others [5]. Nutritional deterioration and altered metabolic regulation frequently coexist with CVD, linking systemic inflammation and body composition changes to increased vulnerability in older adults.

Physical frailty represents a state of dysregulated homeostasis in response to stressors following a dynamic process involving the musculoskeletal system [6].

Various techniques are available to assess body composition, including anthropometry, bone densitometry (DXA), and bioelectrical impedance (BIA). The latter offers a quick, non-invasive, and clinically practical method based on Ohm’s law, deriving impedance (Z) from resistance (R) and reactance (Xc) as (Z^2^ = R^2^ + Xc^2^) [7,8,9].

### 1.1. Body Composition, Frailty, and CVD

Body composition, especially compartmental body composition, has an inverse association with frailty as measured by Fried’s five frailty criteria [10], along with muscle mass and the muscle mass index (*p* < 0.001). In a study of hemodialysis patients, those identified as frail using the Clinical Frailty Score had a higher body mass index (BMI), lower muscle mass, and a higher percentage of body fat [11].

In terms of cardiovascular disease, muscle mass has been identified as a powerful predictor of risk in patients with coronary artery disease (CAD) undergoing cardiac intervention, as well as in patients with heart failure (HF) [12,13]. A reduction in lean body mass (FFM) is a progressive indicator of frailty in patients with cardiovascular disease [14]. A low appendicular skeletal mass index (ASMI) could be used as an independent predictor of major adverse cardiac events and mortality in patients with CAD [15]. Additionally, other studies have indirectly linked bone mineral density to cardiovascular risk in older adults [16].

### 1.2. Phase Angle as a Prognostic Value

In addition to providing compartmental characteristics as described above, bioelectrical impedance analysis (BIA) can evaluate the body’s bioelectrical activity, allowing for the interpretation of cell nutrition levels [1]. The determination of the phase angle (ϕ) measures the quality of the cell membrane and is calculated by performing the arctangent of the reactance divided by the resistance and then multiplying by 180° and dividing by π (ϕ = arctangent (Xc/R) × 180°/π).

Several studies have linked a decrease in phase angle in different groups of patients with syndromes such as geriatric syndrome, frailty, and mortality, although there is no consensus on the specific cut-off points (frailty: men 3.1–5.6°; women 2.7–5.4°) [17,18]. In older adults, the phase angle has been identified as a modifiable risk factor associated with physical performance, improving the prognostic value when the phase angle value increases [19,20].

In hospitalized patients, a cut-off point for the total body phase angle has been identified as a predictor of malnutrition, with 5.3° for women (AUC = 0.815; sensitivity = 0.703; specificity = 0.635) and 5.4° for men (AUC = 0.851; sensitivity = 0.821; specificity = 0.779) [21]. Similarly, in a study conducted with patients in an intensive care unit, those who survived the process had a higher mean phase angle value than those who died, with a cut-off point of 4.6° (AUC = 0.63; 95% CI = 0.58–0.70; sensitivity = 0.080; specificity = 0.049) [22].

The cut-off point identified in a study of hemodialysis patients as a predictive factor for mortality was 4.5° (AUC = 0.754; 95% CI = 0.151–0.346; sensitivity = 0.488; specificity = 0.901). In this study, the most frequent cause of mortality was CVD, and these patients showed a lower phase angle [23].

The phase angle in patients with CVD is smaller than in patients without CVD [24]. In a study by Langer et al., women who developed CVD showed a smaller phase angle (6.3° vs. 6.0°; *p* < 0.001) [25].

In another study, the cut-off point identified as a predictive factor for mortality in patients with acute decompensated heart failure was ≤ 4.9° (AUC = 0.68; 95% CI = 0.62–0.74; sensitivity = 0.75; specificity = 0.44) [26]. In patients undergoing cardiac surgery, a phase angle < 4.5° behaved as a predictive factor for mortality, morbidity, and frailty [27].

Critical transitions between suboptimal states of frailty and high-risk states may require early interventions before frailty is established [6]. Zanforlini et al. estimated that for every 1° increase in phase angle in older adults, the probability of improvement in the frailty state was 4.53 times (95% CI: 1.18–17.46) [19]. Phase angle in CVD can be useful as a tool for monitoring the physiological state of the body in general; however, the use of electrical bioimpedance to determine segmental phase angles can be oriented in clinical practice. States or diseases that do not influence the body in general but do influence certain organs must be taken into account [28]. The technique is fast, portable, and non-invasive, so it could complement the assessment of hospitalized frail patients with CVD [24].

While whole-body phase angle has been linked to mortality and frailty, little is known about the value of segmental measurements in CVD populations. The heterogeneity of cut-off points across studies further limits clinical applicability. Considering that CVD often involves inflammation, segmental analysis could provide more localized information on muscle asymmetry and fluid distribution in relation to the detection of frailty.

For this reason, our goal was to evaluate the prognostic value of segmental phase angles obtained using electrical bioimpedance in identifying physical frailty in hospitalized older adults with CVD. The specific objectives were to assess the association between segmental phase angles and physical frailty status in hospitalized older adults with CVD. We also aimed to determine segmental phase angle cut-offs that discriminate frailty by sex and to assess the relationship between cachexia biomarkers (CRP, hemoglobin, and albumin) and frailty status.

Therefore, we anticipate that segmental phase angles are inversely associated with frailty status in hospitalized older adults with CVD and that sex-specific cut-off points have a diagnostic capacity.

## 2. Materials and Methods

### 2.1. Design, Population, and Sample

A cross-sectional observational study was conducted in a cohort of patients admitted to a conventional cardiology inpatient unit of a tertiary care hospital in the Spanish public health network in the region of Castilla y León between March 2022 and April 2024. The study included conscious and oriented individuals aged 60 years or older who had been diagnosed with coronary artery disease, infective endocarditis, heart failure, arrhythmias, and valvular heart disease according to the cardiovascular disease models defined by scientific societies [29]. A threshold of 60 years old was selected to capture early frailty transitions, as physical decline and muscle mass loss can begin before the age of 65. Patients with previously diagnosed cognitive impairment (ICD-10-CM Diagnosis Code R41.81), severe motor disability that prevented them from standing or required prolonged bed rest, wearers of pacemakers and implantable internal defibrillators (ICDs), and those with a hospital stay of less than 3 days were excluded.

#### Sample Size

The sample size was estimated to assess the predictive capacity of seven segmental phase angles on physical frailty status using binary logistic regression, considering a 30% proportion of events (frailty) in older adults with CVD [30].

Recent methodological recommendations suggest having between 5 and 9 events per variable (EPV) as a suitable criterion for concise models in observational studies [31]. It was determined that between 117 and 158 subjects would be needed to accommodate a model with seven predictors. With a final sample of 157 patients and 45 events (cases with frailty), an EPV of 6.4 was achieved, falling within an acceptable range to ensure model stability and minimize the risk of overfitting. Additionally, this sample size was adequate to detect associations with a minimum expected odds ratio of 2, at a 95% confidence level, and with 80% statistical power.

Patient recruitment occurred consecutively, with individuals signing informed consent upon admission to the cardiology unit during the study period.

The methodological design and writing of this study followed the recommendations outlined in the STROBE statement for cross-sectional observational studies [32].

### 2.2. Variables and Measurements

Sociodemographic data were collected, including biological sex defined at birth, age, and primary diagnosis at admission. Clinical information was obtained directly from the patients’ medical records, including systolic blood pressure (BP; mmHg), diastolic blood pressure (SBP; mmHg), and heart rate (HR; beats per minute).

The length of hospital stay was also identified.

#### 2.2.1. Blood Biomarkers

The results of blood tests during the admission period were collected for total cholesterol (mg/dL), HDL cholesterol (mg/dL), LDL cholesterol (mg/dL), glucose (mg/dL), NT-proBNP (N-terminal pro b-type natriuretic peptide: pg/mL), CRP (C-reactive protein: mg/L), hemoglobin (g/dL), and serum albumin (g/dL).

#### 2.2.2. Frailty

Frailty was determined using the five criteria of the Fried scale [10]:

1. Unintentional weight loss of more than 4.5 kg or more than 5.0% in less than one year.
2. Feeling of general exhaustion (low energy and resistance according to the CES-D depression scale) [33]. Participants were asked: “Do you feel that everything you do takes effort?” and/or “Do you feel that you cannot get going?” A positive response to either question for more than three days in the previous week was considered indicative of exhaustion.
3. Weakness (measured using a Digital Hand Dynamometer).
4. Slow walking speed (time to cover 4.57 m adjusted for gender and height). A trained researcher assessed walking speed measured along the hospital corridor. Patients completed the test independently or with the assistance of a walking aid (cane or walker) when routinely used in daily activities.
5. Weekly physical activity level (determined using the Minnesota Leisure Time Activity Questionnaire (MLTAQ) stratified by gender; men: 383 kcal/week and women: 270 kcal/week) [34].

According to these criteria, patients were classified as follows:

- Frail patients: Three or more of the above criteria were met.
- Patients with pre-frailty: Those who met one or two of the above criteria.
- Patients without frailty: Those who did not present any of the previous criteria.

#### 2.2.3. Cardiovascular Risk

Cardiovascular risk was assessed using the Framingham Risk Assessment Scale, as recommended by the American Heart Association (AHA). The variables considered included age, sex, smoking status (YES/NO), diabetes mellitus (YES/NO), HDL cholesterol, total cholesterol, and systolic and diastolic blood pressure. High risk was defined as a percentage ≥ 20% [35].

#### 2.2.4. Body Composition and Phase Angles

Anthropometric measurements were taken, including height (cm) using a standard stadiometer, body weight (kg) measured with a TANITA MC-780 scale (Arlington Heights, IL, USA), body mass index (kg/m^2^), and abdominal perimeter (cm) measured with ergonomic tape with automatic winding (accuracy 1 mm). Body composition was assessed using eight-point bioimpedance analysis (TANITA MC-780). This technique measures the impedance of the arms, legs, right hemisphere, and left hemisphere at six different frequencies for each body segment (1 kHz, 5 kHz, 50 kHz, 250 kHz, 500 kHz, and 1000 kHz). The frequency used for the analysis in this study was 50 kHz.

The results included metabolic age (an estimation based on basal metabolic rate), total body water (kg); intracellular body water (kg); extracellular body water (kg); body fat percentage; skeletal muscle mass (kg); fat-free body mass (kg); segmental fat percentage (right arm, left arm, right leg, left leg, right hemisphere, and left hemisphere); and segmental PhAs at 50 kHz for the right hemisphere, left hemisphere, right arm, left arm, right leg, left leg, and both legs separately.

The TANITA MC-780 bioimpedance analyzer provides PhA values as negative numbers due to its internal software convention. Since the phase angle is defined as a positive parameter, all analyses in this study were conducted using absolute PhA values. This approach ensures consistency with the literature, where PhA is typically reported as a positive value. This convention is common in multifrequency BIA devices and does not affect comparability with previously published data.

Obesity was defined as BMI ≥ 27 kg/m^2^, based on its correlation with body fat percentage [36].

The sarcopenia index was defined as ASM/height^2^ according to the European Sarcopenia Group (EWGSPO2) (men < 7.0 kg/m^2^; women < 5.5 kg/m^2^) [37] and according to the International Sarcopenia Group (IWGS) (men ≤ 7.23 kg/m^2^; women ≤ 5.67 kg/m^2^) [38].

Sarcopenic obesity was identified by a high percentage of body fat/low grip strength (men: >31% fat/<27 kg strength; women: >43% fat/<16 kg strength) [39].

### 2.3. Procedure

Before beginning data collection, all nurses on the Cardiology Department’s care team underwent specific training in the standardized application of the Fried scale and the data collection protocol to minimize measurement bias. Three nurses received specialized training for the TANITA MC-780 bioimpedance device. Training took place one week prior to the study’s commencement to promote consistency in data collection and reduce interobserver variability.

Upon identifying eligible patients and obtaining informed consent, nurses assessed frailty status using Fried’s five criteria on the third day of hospitalization. This assessment was supplemented by anthropometric measurements (weight, height, waist circumference) after patients signed the informed consent form. The principal investigator and three trained nurses conducted body composition analysis using multifrequency electrical bioimpedance with patients in a standing position. All bioimpedance measurements were taken under standardized conditions: after at least two hours of fasting following a main meal and without prior physical exercise or diuretic administration.

Clinical and analytical data (blood pressure, heart rate, total cholesterol, HDL, LDL, glucose, NT-proBNP, CRP, hemoglobin, and albumin) were gathered from the patients’ electronic medical records, documenting the first determination after hospital admission.

Finally, after collecting all clinical, anthropometric, frailty, and body composition variables, each patient’s cardiovascular risk was calculated by category using the Framingham scale. A value equal to or greater than 20% was considered high risk.

This standardized procedure ensured consistent measurement of all variables over time, guaranteeing that the data accurately reflected the clinical and functional status of the patients upon hospital admission.

### 2.4. Ethical Considerations

All participants signed informed consent in accordance with the Patient Information Sheet template of the Valladolid East Health Area. The anonymity of patients and participant data was maintained at all times using Research Electronic Data Capture (REDCap). The researchers affirm that they follow the bioethical standards outlined in the Declaration of Helsinki; the Oviedo Convention on Human Rights and Biomedicine; and Organic Law 3/2018, of 5 December on the Protection of Personal Data and Guarantee of Digital Rights. The study received approval from the Ethics Committee of the Valladolid East Health Area under PI 20-1612.

### 2.5. Statistical Analysis

All variables described above were analyzed using SPSS 26.0 (IBM, Armonk, New York, NY, USA). Categorical variables were expressed as absolute values and percentages, while continuous variables were expressed as mean ($\bar{X}$) ± standard deviation (SD).

Associations between each variable and the dependent variable (frailty) were examined using the Chi-square test and Fisher’s exact test for qualitative variables. For continuous variables, Student’s *t*-test or the Mann–Whitney U test was applied for those not meeting the normality assumption. Additionally, comparisons across frail, pre-frail, and non-frail groups were performed using ANOVA or the Kruskal–Wallis test for continuous variables and Chi-square tests for categorical variables. We adjusted the *p*-value using Bonferroni correction.

Univariate binary logistic regression was conducted to identify prognostic factors for frailty. The final model calculated adjusted odds ratios (ORs) with 95% confidence intervals for variables with a *p*-value < 0.05.

The model’s goodness of fit was assessed using the Hosmer–Lemeshow test.

Receiver Operating Characteristic (ROC) curves were utilized to determine cut-off points for segmental phase angles, followed by evaluation of the sensitivity, specificity, and likelihood ratio using the Fagan nomogram [40]. A sensitivity analysis was also performed, excluding patients with infective endocarditis, to assess the robustness of the results.

No missing data were present for the main variables, and checklists were employed for all analyses.

## 3. Results

### 3.1. Characteristics of the Study Participants

**Figure 1:**
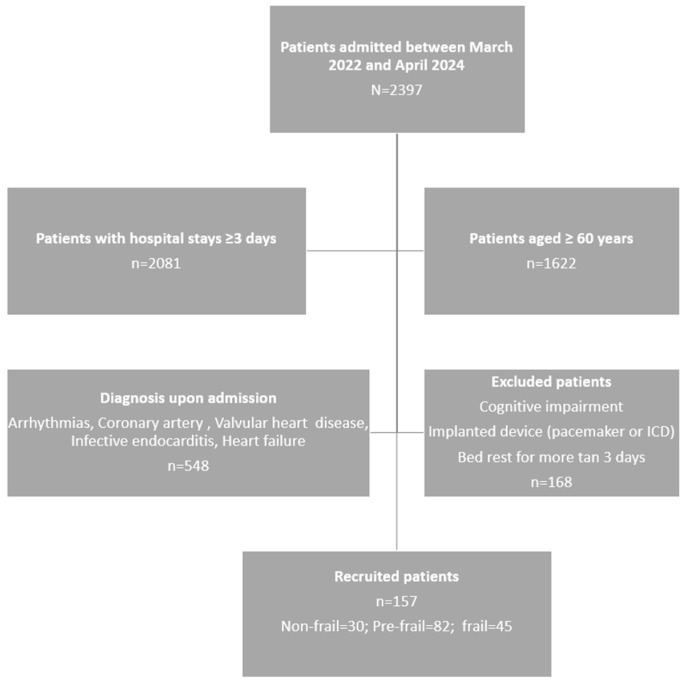
Patient selection flowchart.

Figure 1 displays the flowchart of the patient’s inclusion process. Out of the 794 patients initially evaluated, 157 were ultimately included based on the defined inclusion and exclusion criteria.

The mean age of the patients studied was 73.23 years (SD = 7.91); 66.24% were men, and 33.76% were women, as shown in Table 1. The majority (75.80%) were between 60 and 79 years old. The overall prevalence of frailty was 28.66%, slightly higher in women (30.19%) than in men (27.88%), although not statistically significant (*p* = 0.091). The most frequent diagnosis was coronary artery disease (61.15%).

In terms of comorbidities, 35.03% had previous diabetes mellitus, and 19.75% had high cardiovascular risk (≥20% according to Framingham), which was more frequent in men (25.00% vs. 9.43%; *p* = 0.021). Patients with diabetes showed statistically lower values in the phase angle of the right leg (*p* = 0.034), left leg (*p* = 0.021), and both legs (*p* = 0.026).

Hospital stays were longer among frail patients (*p* < 0.01) and men than among women (*p* = 0.043). Frail men had particularly long stays (*p* = 0.011).

Regarding nutritional and biochemical indicators, 56.05% were obese (BMI > 27 kg/m^2^), with no significant differences between sexes. Given the high prevalence of obesity, exploratory analyses were performed to examine whether these conditions influenced segmental phase angle values. We did not observe significant differences when stratified by BMI categories. Women showed significantly higher levels of total cholesterol, HDL, and LDL (*p* < 0.05 in all cases). Mean hemoglobin was lower in frail patients (*p* = 0.032), with a higher proportion of Hb < 12 g/dL. This difference was particularly significant among men with frailty (*p* = 0.049) but not observed in women. Anemia (Hb < 12 g/dL) was present in 16.60% of patients, which was more prevalent in the frail group. CRP was higher in frail patients, although not statistically significant.

### 3.2. Body Composition and Frailty

Table 2 summarizes the main body composition and functional parameters according to frailty status. The average metabolic age of the sample was 63.76 years (SD = 11.14) and was significantly higher in patients with frailty (*p* = 0.001), for both men and women (*p* = 0.002). The average percentage of body fat was 27.20% (SD = 7.66), and the sarcopenia index was 8.17 kg/m^2^ (SD = 1.23). While patients with frailty exhibited higher values of total and segmental fat, these differences did not reach statistical significance overall. Details of data by sex and other parameters are available in Supplementary Table S1.

The average waist circumference was 102.64 cm (SD = 13.33). In the analysis by sex and frailty, robust men had a significantly smaller waist circumference (<100 cm) than pre-frail and frail men (*p* = 0.029 and *p* = 0.033).

Analysis by sex revealed that 13.50% of men had a body fat percentage above 31%, while only 3.77% of women had a percentage above 43%. Sarcopenic obesity, defined as an elevated body fat percentage combined with reduced grip strength, was identified in 6.63% of men, but not in women. Additionally, the percentage of segmental fat in the right arm was significantly higher in individuals with frailty (*p* = 0.018).

The sarcopenia index (ASM/height^2^) showed values within the normal range in all groups, according to both the EWGSOP2 and IWGS criteria. Although frail patients exhibited slightly higher values than pre-frail and robust patients, no clinically relevant sarcopenia was observed.

Handgrip strength in the dominant hand was significantly greater in robust patients than in pre-frail and frail patients (*p* < 0.001). This difference was consistent in both men and women (*p* = 0.000; *p* = 0.001), confirming the association between functional frailty and decreased muscle strength.

In addition to body fat percentage, other indicators of body composition were evaluated. The average percentage of total body water was 51.78% (SD = 5.79), with lower values in patients with frailty, although without significant differences. Fat-free mass was 54.04 kg (SD = 11.40), and average skeletal muscle mass was 51.32 kg (SD = 10.86), with higher values in men.

### 3.3. Relationship Between Segmental Phase Angles and Frailty Status

Statistically significant differences were observed between segmental phase angles and clinical diagnoses at admission (Table 3). The mean of most phase angles was higher in patients with coronary artery disease, except in the left and right hemispheres and both arms, where the highest values were observed in cases of infective endocarditis (*p* < 0.01) (Supplementary Table S2).

In the overall sample analysis, segmental phase angles showed mean values greater than 5° on the right and left sides of the body, as well as in both arms (detailed mean ± SD values and post hoc Bonferroni results are provided in Supplementary Table S2). Patients with frailty consistently displayed lower phase angle values in all segments. These differences were statistically significant compared with the pre-frailty group and, in the case of the left arm, the non-frail group (*p* = 0.011). However, no significant differences were observed in the phase angle of the right side of the body when comparing frailty groups (Table 4).

Analysis by sex revealed that women consistently had lower phase angle values in both frail and non-frail individuals than men. In the male group, statistically significant differences were found between frail and pre-frail individuals in all segments except the right half of the body. By contrast, only in women did this angle show significant differences between frailty groups (*p* = 0.045) (detailed mean ± SD values and post hoc Bonferroni results are provided in Supplementary Table S3).

### 3.4. Relationship of Segmental Phase Angles and Cachexia Biomarkers with Frailty Status

In the univariate logistic regression analysis, all segmental phase angles were significantly associated with frailty status. Each additional degree of phase angle increased the odds of developing frailty, with the left arm phase angle showing the strongest association (OR = 2.30; 95% CI: 1.42–3.72; *p* = 0.001), followed by the left leg phase angle (OR = 1.95; 95% CI: 1.34–2.83; *p* = 0.001) and the left hemisphere phase angle (OR = 2.00; 95% CI: 1.26–3.16; *p* = 0.003). CRP levels greater than 5 mg/L were related to frailty status (*p* = 0.030), as was hemoglobin < 12 g/dL (*p* = 0.011) (Table 5).

### 3.5. Analysis of ROC Curves and Cut-Off Points for Segmental Phase Angles

**Figure 2:**
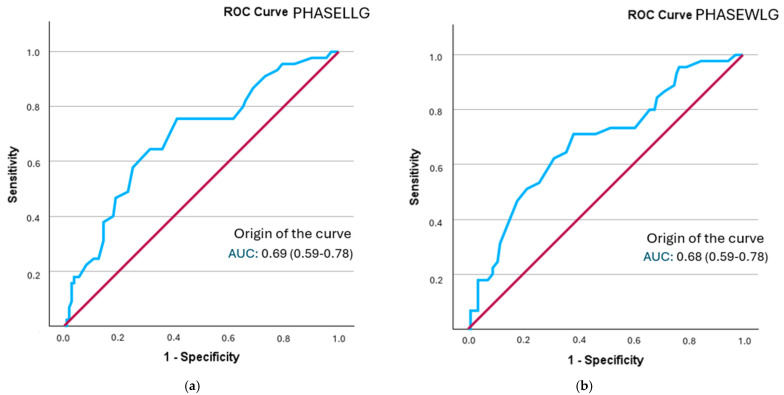
A comparison of ROC curves for segmental phase angles ((**a**): PHASELLG; (**b**): PHASEWLG) across the entire sample in relation to frailty status. Note: PHASE°LLG: phase angle of the left leg; PHASE°WLG: phase angle of both legs.

The analysis of the ROC curve revealed that all segmental phase angles demonstrated a statistically significant discriminatory ability in identifying frailty status. Optimal cut-off points were established for each segment, and sensitivity, specificity, and positive likelihood ratio (PLR) values were computed. The best model was that of the phase angle of the left leg, followed by both legs, with a performance close to 0.7 (Figure 2). Additionally, the posterior probability was determined using the Fagan nomogram.

The phase angle with the highest sensitivity was found in both legs (0.694), although the positive likelihood ratio was lower than that of the phase angle of the right leg (1.79) (Supplementary Figure S1). The probability of increased frailty following the determination of the phase angle rose from 28.66% to 36% in both legs and to 42% in the right leg (refer to Table 6).

Sensitivity analyses were conducted, excluding the two patients diagnosed with infective endocarditis, given the difference in mean phase angle values compared with the other diagnoses. The resulting AUC values (0.67–0.69), likelihood ratios (1.45–2.01), and post-test probabilities (36–44%) were almost identical to those of the complete sample. The most discriminatory segments, the left leg and the left body, remained unchanged, confirming that these atypical cases did not significantly influence the diagnostic performance or overall conclusions of the study.

**Figure 3:**
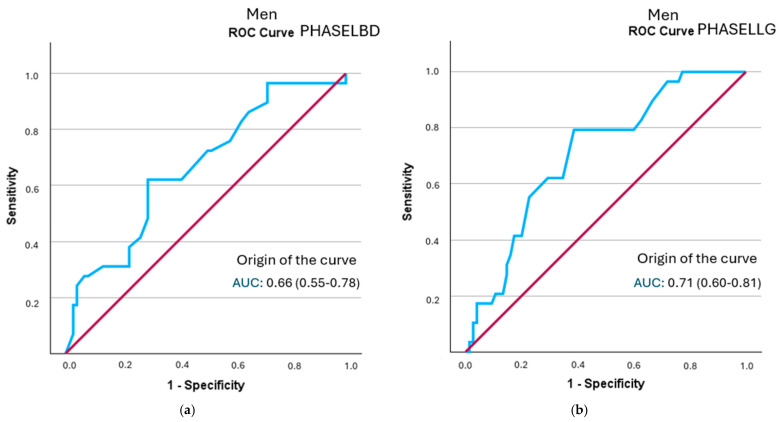
A comparison of ROC curves of segmental phase angles ((**a**): PHASELBD; (**b**): PHASELLG) versus frailty status in men. Note: PHASE°LBD: phase angle of the left half of the body; PHASE°LLG: phase angle of the left leg.

In the sex-specific analysis, all segmental phase angles in men showed statistically significant discriminatory ability for frailty status. The left hemisphere phase angle had the highest positive likelihood ratio (LR+ = 2.12; 95% CI: 1.35–3.33), followed by the left leg angle (LR+ = 2.05; 95% CI: 1.46–2.88) (Figure 3 and Table 7). All curves are represented in Supplementary Figure S2.

**Figure 4:**
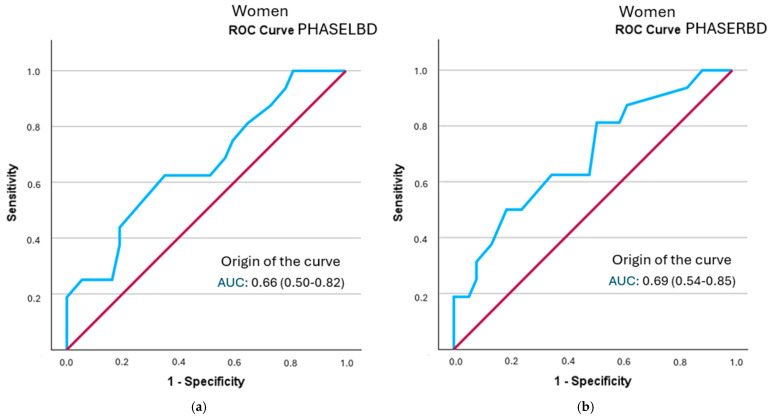
A comparison of ROC curves of segmental phase angles ((**a**): PHASELBD; (**b**): PHASERBD) versus frailty status in women. Note: PHASE°LBD: phase angle of the left half of the body; PHASE°RBD: phase angle of the right half of the body.

In the female-specific analysis, several segmental phase angles did not reach statistical significance in relation to frailty status: right leg (*p* = 0.078), left leg (*p* = 0.056), right arm (*p* = 0.108), and left arm (*p* = 0.106). The cut-off points obtained were lower than those identified in men. None of the evaluated segments showed clinically relevant individual diagnostic utility (LR+ > 2). Detailed results are shown in Table 8 and Figure 4 (all curves are represented in Supplementary Figure S3).

## 4. Discussion

This is one of the first studies to explore sex-specific cut-off values for segmental phase angles in hospitalized CVD patients. Frailty was present in more than one in four hospitalized older adult patients with CVD. This proportion was higher in women than in men, consistent with previous studies that have shown female sex to be associated with greater functional vulnerability and risk of frailty [41]. This difference may be linked to a higher prevalence of comorbidities, lower relative muscle mass, and lower functional reserve in women. However, these factors should be interpreted cautiously due to the observational design of the present study.

Metabolic age was significantly higher in patients with frailty, mirroring the trend seen in chronological age. This alignment has been noted before [4] and reflects the cumulative functional impact of aging and multimorbidity. Some authors have even suggested that metabolic age could serve as a better indicator of cardiovascular risk than chronological age [42].

Cut-off points were identified for segmental phase angles associated with frailty status. The phase angle of the left hemisphere (5.15°) and the left leg (4.45°) showed the highest diagnostic yield in men, with positive likelihood ratios greater than 2, indicating moderate predictive value. The posterior probability of frailty in these segments was 45%. Similarly, the phase angle of the left leg, with a cut-off point of 4.45°, also showed fair predictive value, with a probability of 44%. In women, after ROC curve analysis, only the cut-off points for the left hemisphere, both legs, and the right hemisphere were statistically significant enough to establish cut-off points. However, the likelihood ratio for determining prognostic value did not exceed 2 points in any of them, limiting its use as the sole discriminant test in this subgroup. The cut-off points identified by other authors are below 5.5° in both men and women, demonstrating the relationship between morbidity and mortality with lower phase angle values [22,23,27].

The cut-off points identified in our study (4.25–5.75°) align with those reported in previous studies involving hospitalized or cardiovascular populations. Stellingwerf et al. [22] reported a phase angle threshold of 4.6° associated with mortality in adult ICU patients, while Yuanzhao et al. [23] found a similar value (4.5°) predicting mortality in hemodialysis patients. Likewise, Mullie et al. [27] identified a phase angle below 4.5° as an independent predictor of 12-month mortality after cardiac surgery. These findings demonstrate that values below approximately 5° reflect clinically significant physiological impairment across diverse clinical contexts. Although the segmental cut-off points proposed for PhA demonstrated moderate predictive value, the modest overall increase in post-test probability indicates that this should be interpreted with caution. These thresholds may be influenced by specific population characteristics, suggesting that segmental phase angles should be considered supportive indicators rather than independent diagnostic tools until more comprehensive validation is available. The disparity observed between sexes could be related to the differences in body composition, distribution of muscle and fat mass, or inflammatory response, factors that also influence the measurement of the phase angle.

The association between lower segmental phase angles and frailty was consistent across all segments analyzed, with the left arm showing the strongest relationship (OR, 2.29 per degree). This association has also been documented in previous studies, which indicated that low phase angle values were significantly associated with a higher risk of frailty in older adults [18]. The present segmental analysis adds a novel perspective not commonly found in clinical studies and enables the detection of topographical differences that could reflect regional alterations in active cell mass or tissue fluid balance. This disaggregation could be useful for future individualized functional assessment strategies, especially in patients with cardiovascular disease, in whom the distribution of the inflammatory load or catabolism may not be homogeneous. The differences between bioimpedance models and the segmental approach used in this study may partly explain the variability in cut-off points reported by other authors [17,43].

An extended discussion of body parameters (BMI, sarcopenia index, fat distribution, and sarcopenic obesity) is provided in Supplementary Discussion S1. In brief, patients with frailty showed higher total and segmental fat percentages, while lean mass parameters remained within the normal range, suggesting that adiposity may play a more prominent role than muscle depletion in this hospitalized cohort.

In the analysis of clinical biomarkers, a higher proportion of patients with low hemoglobin levels (<12 g/dL) was observed in the frailty group, as well as lower mean values, with statistical significance. This finding is consistent with what has been described in the literature, where anemia has been identified as a functional marker associated with frailty and clinical deterioration in older patients with cardiovascular disease [6]. C-reactive protein, as a marker of systemic inflammation, also showed a significant association with frailty status, in line with the hypothesis that chronic inflammatory processes contribute to progressive functional decline. We must bear in mind that the acute cardiological process itself may be the cause of elevated CRP, and so, in subsequent studies, it may be advisable to stratify levels and assess the evolution from admission to discharge. However, serum albumin levels, although lower in frail patients, did not reach statistical significance, which could be due to the lower sensitivity of this marker in early stages of nutritional deterioration or the influence of other clinical variables.

These results reinforce the role of frailty as a complex biological state in which low-grade inflammation, hematological dysregulation, and altered body composition coexist and interact with each other. Authors such as Fernández-Jiménez et al. and Korzonek-Szlacheta et al. [14,21] have pointed out that integrating clinical biomarkers with functional and structural parameters, such as segmental phase angles, can enhance the identification of vulnerable patients and predict adverse outcomes. This multidimensional approach is particularly valuable in hospitalized older adults with CVD, where clinical presentations are frequently intricate and dynamic.

Our findings are consistent with previous studies in hospital settings where bioimpedance parameters, such as phase angle, were useful in critically ill patients admitted to intensive care units, supporting their role as prognostic markers beyond nutritional assessment [44]. Similarly, in patients undergoing elective cardiac surgery, lower preoperative phase angles have been independently associated with frailty, impaired cardiac function, increased fluid requirements, and longer ICU stays, highlighting the prognostic value of phase angle in the cardiovascular population [45]. The ability of bioimpedance to detect regional alterations in body composition, acting as a versatile and non-invasive tool, favors its integration into routine hospital protocols, especially in cardiology wards, where early functional decline often goes unnoticed.

The use of BIA in patients with hospital stays shorter than 3 days should be evaluated in terms of practicality, cost-effectiveness, and potential clinical utility in future studies.

Segmental PhA may support frailty screening in men hospitalized with CVD. However, the limited predictive value in women underscores the need for sex-specific validation. Furthermore, combining segmental phase angle assessment with biomarkers such as hemoglobin or CRP may enhance real-time frailty detection and facilitate timely multidisciplinary interventions. Future studies should confirm these findings in larger samples, incorporate multifactorial predictive models, and explore the longitudinal behavior of segmental phase angles in response to nutritional or physical interventions, as well as their prognostic value for cardiovascular events and clinically relevant outcomes such as readmission, functional decline, and mortality.

From a clinical perspective, the implementation of segmental bioimpedance assessment is feasible within standard hospital workflows. Measurement can be performed by trained nurses during the first days of hospitalization, in parallel with functional and nutritional assessment using a multidisciplinary approach. This method is low-cost, time-efficient, and could be incorporated into a simple algorithm to support early frailty detection and optimize care pathways.

### Strengths and Limitations

This study has several strengths, including the standardized application of the measurement protocol by trained personnel; the use of an accessible and reproducible multifrequency segmental bioelectrical impedance technique; and stratified analysis by sex and clinical biomarkers, allowing for a more detailed interpretation.

However, the study’s observational, cross-sectional design prevents the establishment of causal relationships between segmental phase angles and frailty status. Additionally, the study was conducted using a single hospital sample, which was limited in size and representativeness, potentially restricting the generalization of the results to other clinical or population settings.

The absence of prospective follow-up prevents us from determining whether phase angle changes predict clinical outcomes such as readmission, functional decline, and mortality. However, the use of standardized diagnostic criteria and the inclusion of various cardiovascular conditions improve the potential applicability of our results to similar cardiology units in other healthcare systems.

Furthermore, while multifrequency segmental bioimpedance, a validated, non-invasive technique, was utilized, its accuracy can be influenced by factors such as hydration status, body position, and inter-device variability, especially in patients with cardiovascular comorbidities. We must bear in mind the possible influence of dynamic fluid shifts during hospitalization, especially in patients receiving diuretic therapy or presenting with peripheral edema. Although measurements were performed under standardized conditions, variations in hydration status and extracellular water distribution may have affected phase angle values. Additionally, reference methods like DXA or computed axial tomography were not employed to validate the body composition values obtained.

Similarly, the analysis by sex revealed a lower predictive capacity of phase angles in women, which may be attributed to a smaller sample size in this subgroup and potential uncontrolled physiological differences or measurement biases. We should note that no sample size calculation was performed by sex.

These limitations should be considered when applying our findings to broader clinical settings. We recommend conducting future multicenter studies to confirm the generalization of these results.

## 5. Conclusions

The results of this study indicate that segmental phase angles obtained through bioimpedance have moderate prognostic value in identifying physical frailty status in hospitalized older adults with cardiovascular disease, particularly in men. The phase angles of the left arm and left leg displayed the highest discriminatory power, with likelihood ratios exceeding 2 and posterior probabilities exceeding 40%. However, in women, diagnostic performance was more limited, suggesting the necessity of utilizing combined approaches.

Segmental analysis offers unique information in comparison to global analysis, enabling the recognition of regional patterns associated with the distribution of active cell mass and body fat. Incorporating segmental analysis into clinical practice, alongside functional tools and biomarkers, has the potential to enhance the early detection of frailty and guide personalized interventions for hospitalized older adults with CVD.

## Figures and Tables

**Table 1:** Clinical and demographic characteristics of participants stratified by sex and frailty status.

Variable	Total (n = 157)	Men (n = 104)	Women (n = 53)	Frail (n = 45)	Pre-Frail (n = 82)	Non-Frail (n = 30)	*p*-Value (Frailty)	*p*-Value (Sex)
Age (years), mean (SD)	73.23 (7.91)	73.42 (7.84)	72.92 (8.10)	**77.98 (7.98)**	71.32 (7.29)	71.47 (6.54)	**<0.001**	0.691
Frailty (%)	28.66	27.88	30.19	—	—	—	—	0.741
Coronary artery disease (%)	61.15	62.50	58.49	—	—	—	—	0.651
Diabetes mellitus (%)	35.03	37.50	30.19	—	—	—	—	0.355
Cardiovascular risk ≥ 20% (%)	19.75	**25.00**	9.43	8.00	16.00	7.00	0.256	**0.021**
Hospital stays (days), mean (SD)	8.72 (5.44)	**9.43 (5.99)**	7.30 (3.82)	**11.35 (6.82)**	7.81 (4.30)	7.30 (4.82)	**<0.01**	**0.043**
BMI > 27 kg/m^2^ (%)	56.05	58.65	50.94	60.00	56.10	50.00	0.568	0.341
Total cholesterol (mg/dL), mean (SD)	160.59 (44.19)	**150.89 (43.21)**	**179.62 (40.03)**	156.53 (45.70)	162.76 (44.34)	160.77 (42.48)	0.608	**<0.001**
HDL (mg/dL), mean (SD)	45.34 (13.69)	**42.53 (11.75)**	**50.85 (15.57)**	42.40 (9.78)	46.49 (15.66)	46.60 (12.64)	0.224	**<0.001**
LDL (mg/dL), mean (SD)	91.59 (36.51)	**85.73 (36.76)**	**103.08 (33.46)**	91.40 (36.85)	91.94 (36.72)	90.90 (36.67)	0.964	**<0.01**
Hb < 12 g/dL (%)	16.56	16.65	16.98	**28.89**	10.98	13.33	**0.032**	0.968
Albumin < 3.2 g/dL (%)	8.92	11.54	3.77	13.33	7.32	6.67	0.231	0.112
CRP > 5 mg/L (%)	48.41	49.04	47.17	62.22	43.90	40.00	0.060	0.833

Note: Significant differences between groups are indicated in bold (*p* < 0.05); BMI: body mass index; Hb: hemoglobin; HDL/LDL: lipoproteins; CRP: C-reactive protein. *p*-value (frailty): comparison between the three frailty groups using ANOVA/Kruskal–Wallis or Chi-square according to the variable. *p*-value (sex): comparison between men and women. Bonferroni-adjusted *p*-value.

**Table 2:** Body composition and functional parameters according to frailty status.

Variable	Frail (n = 45)	Pre-Frail (n = 82)	Non-Frail (n = 30)	*p*-Value
Metabolic age (years)	69.42 ± 9.44	62.15 ± 10.56	59.66 ± 8.96	**0.001**
% Body fat	29.81 ± 6.98	26.89 ± 8.01	25.27 ± 6.93	0.068
Abdominal perimeter (cm)	106.52 ± 12.91	103.12 ± 13.31	96.90 ± 11.12	**0.027**
% Total body water	49.74 ± 6.21	52.12 ± 5.38	53.04 ± 5.36	0.062
Fat-free mass (kg)	50.94 ± 10.73	54.46 ± 11.35	57.13 ± 10.63	0.075
Muscle mass (kg)	48.25 ± 10.12	51.79 ± 10.89	54.56 ± 10.02	0.082
% Right arm fat	33.91 ± 7.45	31.03 ± 7.96	29.63 ± 7.31	**0.018**
Grip strength (kg)	20.05 ± 8.09	26.25 ± 9.70	31.53 ± 7.59	**<0.001**
Sarcopenia obesity (%)	8.89	2.44	0.00	**0.047**

Note: Values are expressed as mean ± SD or percentage. Values with statistically significant differences (*p* < 0.05) are highlighted in bold (Bonferroni-adjusted).

**Table 3:** Pairwise comparisons of mean segmental phase angles between diagnoses upon admission.

Segmental Phase Angle	Diagnoses	*p*-Value
PHASE°LBD (Left-Half Body)	Infective Endocarditis > Arrhythmias	0.011
	Infective Endocarditis > Heart Failure	0.001
	Infective Endocarditis > Coronary artery disease	0.019
	Infective Endocarditis >Valvopathies	<0.001
	Coronary artery disease > Heart Failure	0.015
	Coronary artery disease > Valvopathies	0.001
PHASE°RRG (Right Leg)	Coronary artery disease > Heart Failure	0.004
	Coronary artery disease > Valvopathies	0.026
	Coronary artery disease > Arrhythmias	0.004
PHASE°LLG (Left Leg)	Coronary artery disease > Heart Failure	0.006
	Coronary artery disease > Valvopathies	0.009
PHASE°RAM (Right Arm)	Infective Endocarditis > Arrhythmias	<0.001
	Infective Endocarditis > Heart Failure	<0.001
	Infective Endocarditis > Coronary artery disease	0.001
	Infective Endocarditis > Valvopathies	<0.001
	Coronary artery disease > Heart Failure	0.007
PHASE°LAM (Left Arm)	Coronary artery disease > Valvopathies	0.006
PHASE°WLG (Both Legs)	Coronary artery disease > Heart Failure	0.003
	Coronary artery disease > Valvopathies	0.007
PHASE°RBD (Right-Half Body)	Infective Endocarditis > Arrhythmias	<0.001
	Infective Endocarditis > Heart Failure	<0.001
	Infective Endocarditis > Coronary artery disease	<0.001
	Infective Endocarditis > Valvopathies	<0.001
	Coronary artery disease > Valvopathies	0.019
	Coronary artery disease > Heart Failure	0.006

Note: *p*-value (Bonferroni-adjusted).

**Table 4:** Mean segmental phase angles by frailty status and sex.

Segmental Phase Angle	Frail (n = 45)	Pre-Frail (n = 82)	Non-Frail (n = 30)	*p*-Value	Sex (by Frailty Status)	*p*-Value
PHASE°LBD (Left-half body)	4.82 ± 0.98	5.23 ± 0.74	5.30 ± 0.80	0.009	Male	0.045
PHASE°RRG (Right leg)	4.13 ± 1.05	4.65 ± 0.76	4.81 ± 1.14	0.002	Male	0.018
PHASE°LLG (Left leg)	4.05 ± 1.00	4.56 ± 0.84	4.78 ± 1.06	<0.001	Male	0.004
PHASE°RAM (Right arm)	5.46 ± 1.12	5.77 ± 0.59	5.84 ± 0.72	0.044	Male	0.004
PHASE°LAM (Left arm)	5.21 ± 0.76	5.76 ± 0.80	5.70 ± 0.81	<0.05	-	n.s.
PHASE°WLG (Both legs)	4.20 ± 1.00	4.77 ± 0.76	4.89 ± 1.07	0.001	Male	0.008
PHASE°RBD (Right-half body)	4.96 ± 1.27	5.29 ± 0.63	5.34 ± 0.78	n.s.	Female	0.045

Note: Values are mean ± SD. n.s.: not significant. *p*-value (Bonferroni-adjusted).

**Table 5:** Relationship of segmental phase angles and cachexia biomarkers with frailty status.

	Standard Error	df	*p*-Value	Odds Ratio	95% CI	
					Lower	Superior
PHASE°LBD	0.24	1	0.003	**2.00**	1.26	3.16
PHASE°RRG	0.18	1	0.001	**1.78**	1.25	2.54
PHASE°LLG	0.19	1	0.001	**1.95**	1.34	2.83
PHASE°RAM	0.23	1	0.015	**1.76**	1.12	2.78
PHASE°LAM	0.25	1	0.001	**2.30**	1.42	3.78
PHASE°WLG	0.19	1	0.001	**1.90**	1.30	2.76
PHASE°RBD	0.22	1	0.025	**1.65**	1.07	2.56
CRP > 5 mg/L	0.36	1	0.030	**0.46**	0.22	0.93
Albumin < 3.2 g/dL	0.57	1	0.232	0.51	0.17	1.55
Hemoglobin < 12 g/dL	0.44	1	0.011	**0.32**	0.14	0.77

Note: PHASE°LBD: phase angle of the left half of the body; PHASE°RRG: phase angle of the right leg; PHASE°LLG: phase angle of the left leg; PHASE°RAM: phase angle of the right arm; PHASE°LAM: phase angle of the left arm; PHASE°WLG: phase angle of both legs; PHASE°RBD: phase angle of the right half of the body; CRP: C-reactive protein. CI: confidence interval. Univariate analysis with frailty as the dependent variable (binary logistic model). Statistically significant associations (*p* < 0.05) are highlighted in bold within the text.

**Table 6:** Prognostic value of segmental phase angles for frailty status based on cut-off points in the total sample.

Segmental Phase Angle	Cut-Off Point °	Sensitivity	Specificity	LR+ (95%CI)	Post-Test Probability % (95% CI)
PHASE°LBD	5.15	0.667	0.607	1.70 (1.25–2.31)	41 (33–48)
PHASE°RRG	4.45	0.689	0.616	1.79 (1.32–2.44)	42 (35–50)
PHASE°LLG	4.25	0.644	0.688	2.06 (1.45–2.93)	45(37–54)
PHASE°RAM	5.75	0.644	0.571	1.50(1.11–2.04)	38(31–45)
PHASE°LAM	5.65	0.689	0.509	1.40 (1.07–1.84)	36(30–43)
PHASE°WLG	4.45	0.694	0.536	1.39 (1.03–1.86)	36(29–43)
PHASE°RBD	5.25	0.644	0.536	1.39(1.03–1.86)	36(29–43)

Note: PHASE°LBD: left half-body phase angle; PHASE°RRG: right leg phase angle; PHASE°LLG: left leg phase angle; PHASE°RAM: right arm phase angle; PHASE°LAM: left arm phase angle; PHASE°WLG: both legs phase angle; PHASE°RBD: right half-body phase angle. LR+: positive likelihood ratio. Posterior probability calculated with a prior prevalence of 28.66% using the Fagan nomogram.

**Table 7:** Prognostic value of segmental phase angles for frailty status according to cut-off points in pen.

Segmental Phase Angle Male	(Cut-Off Point) °	Sensitivity	Specificity	LR + (95%CI)	Post-Test Probability % (95% CI)
PHASE°LBD	5.15	0.621	0.707	2.12 (1.35–3.33)	45(34–56)
PHASE°RRG	4.45	0.690	0.573	1.62 (1.13–2.31)	39(30–47)
PHASE°LLG	4.45	0.793	0.613	2.05 (1.46–2.88)	44(36–53)
PHASE°RAM	5.95	0.655	0.533	1.40 (0.98–2.01)	35(27–44)
PHASE°LAM	6.05	0.828	0.480	1.59(1.21–2.09)	38(32–45)
PHASE°WLG	4.55	0.724	0.613	1.87 (1.30–2.69)	42(33–51)
PHASE°RBD	5.65	0.793	0.427	1.38 (1.06–1.81)	35(29–41)

Note: PHASE°LBD: phase angle of the left half of the body; PHASE°RRG: phase angle of the right leg; PHASE°LLG: phase angle of the left leg; PHASE°RAM: phase angle of the right arm; PHASE°LAM: phase angle of the left arm; PHASE°WLG: phase angle of both legs; PHASE°RBD: phase angle of the right half of the body. LR+: positive likelihood ratio. Posterior probability calculated according to the prevalence of frailty in men.

**Table 8:** Prognostic value of segmental phase angles for frailty status based on cut-off points in women.

Segmental Phase Angle Female	Cut-Off Point °	Sensitivity	Specificity	LR + (95%CI)	Post-Test Probability % (95% CI)
PHASE°LBD	4.65	0.625	0.649	1.78 (1.00–3.18)	43(30–58)
PHASE°WLG	4.55	0.668	0.622	1.82 (1.07–3.08)	44(32–57)
PHASE°RBD	5.15	0.812	0.486	1.58 (1.07–2.34)	41(32–50)

Note: PHASE°LBD: phase angle of the left half of the body; PHASE°WLG: phase angle of both legs; PHASE°RBD: phase angle of the right half of the body. LR+: positive likelihood ratio. The posterior probability is calculated based on the prevalence of frailty in women.

## Data Availability

The data presented in this study are available upon request from the corresponding author. The data are not publicly available due to privacy or ethical restrictions.

## Supplementary Materials

The following supporting information can be downloaded at https://www.mdpi.com/article/10.3390/healthcare13212816/s1: Table S1: Detailed body composition parameters, sarcopenia, and sarcopenic obesity vary according to sex and frailty status. Table S2: Detailed comparison between clinical diagnosis upon admission and segmental phase angles. Table S3. Detailed comparison between frailty status, sex, and segmental phase angles. Figure S1. A comparison of ROC curves of segmental phase angles in the overall sample versus frailty status. Figure S2. A comparison of ROC curves of segmental phase angles in the overall sample versus frailty status in men. Figure S3. A comparison of ROC curves of segmental phase angles in the overall sample versus frailty status in women. Discussion S1: Detailed analysis of body composition, adiposity patterns, and sarcopenia in relation to frailty status. Reference [46] appears in Supplementary Discussion S1.

## Abbreviations

The following abbreviations are used in this manuscript:

ASM	Appendicular skeletal mass
BIA	Bioelectrical impedance
BMI	Body mass index
CRP	C-reactive protein
CVD	Cardiovascular disease
DXA	Bone densitometry
FFM	Fat-free mass
EWGSOP2	European Working Group on Sarcopenia in Older People (2nd definition)
IWGS	International Working Group on Sarcopenia
NT-ProBNP	N-terminal pro b-type natriuretic peptide
PhA	Phase angle
PHASE°LBD	Phase angle of the left half of the body
PHASE°RRG	Phase angle of the right leg
PHASE°LLG	Phase angle of the left leg
PHASE°RAM	Phase angle of the right arm
PHASE°LAM	Phase angle of the left arm
PHASE°WLG	Phase angle of both legs
PHASE°RBD	Phase angle of the right half of the body
