# Supplementary material for: Segmental Bioimpedance Phase Angles for Frailty Detection in Hospitalized Older Adults with Cardiovascular Disease: A Cross-Sectional Observational Study
Source: Healthcare (Basel). 2025 Nov 6;13(21):2816. doi: 10.3390/healthcare13212816 (PMC12610249; doi:10.3390/healthcare13212816)
Supplement: Supplementary file 1 [file healthcare-13-02816-s001.zip › healthcare-3921916-supplementary.pdf]

Supplementary Table S1. Detailed body composition parameters, sarcopenia, and sarcopenic obesity vary according to sex and frailty status.

Variable	Total (n = 157)	Male (n = 104)	Female (n = 53)	Frail (n = 45)	Pre-frail (n = 82)	Non-frail (n = 30)	p-value (frailty)	p-value (sex)
Metabolic age (years), mean (SD)	63.76 (11.14)	62.91 (11.59)	65.45 (10.16)	**69.42 (9.44)**	62.15 (10.56)	59.66 (8.96)	**0.001**	0.102
% Body fat, mean (SD)	27.20 (7.66)	25.72 (6.63)	30.28 (8.73)	29.81 (6.98)	26.89 (8.01)	25.27 (6.93)	0.068	**<0.01**
Abdominal perimeter (cm), mean (SD)	102.64 (13.33)	**104.83 (13.27)**	98.49 (12.36)	106.52 (12.91)	103.12 (13.31)	**96.90 (11.12)**	**0.027**	**0.003**
% Total body water, mean (SD)	51.78 (5.79)	**52.84 (5.43)**	49.67 (5.96)	49.74 (6.21)	52.12 (5.38)	53.04 (5.36)	0.062	**<0.01**
Fat-free mass (kg), mean (SD)	54.04 (11.40)	**59.80 (8.74)**	42.17 (7.19)	50.94 (10.73)	54.46 (11.35)	57.13 (10.63)	0.075	**<0.001**
Muscle mass (kg), mean (SD)	51.32 (10.86)	**56.79 (8.32)**	39.75 (6.79)	48.25 (10.12)	51.79 (10.89)	54.56 (10.02)	0.082	**<0.001**
ASM/height^2 (kg/m^2), mean (SD)	8.17 (1.23)	**8.61 (1.09)**	7.33 (1.00)	8.31 (1.17)	8.10 (1.27)	8.17 (1.18)	0.404	**<0.001**
% ASM/body weight, mean (SD)	17.13 (2.80)	**17.56 (2.41**)	16.24 (3.36)	16.85 (2.75)	17.13 (2.87)	17.66 (2.64)	0.339	**<0.05**
% Right arm fat, mean (SD)	31.42 (7.84)	29.84 (6.90)	**34.63 (8.45)**	**33.91 (7.45)**	31.03 (7.96)	29.63 (7.31)	**0.018**	**<0.01**
Grip strength (kg), mean (SD)	25.34 (9.72)	**29.04 (8.45)**	17.70 (6.20)	**20.05 (8.09)**	26.25 (9.70)	**31.53 (7.59)**	**<0.001**	**<0.001**
Sarcopenic obesity (%)	4.46	**6.63**	0.00	8.89	2.44	0.00	**0.047**	**0.041**

Note: ASM, appendicular skeletal mass; sarcopenic obesity is defined as a combination of high % segmental fat + and low grip strength (Benz et al., 2024). The p-value for frailty indicates a comparison between frail, pre-frail, and non-frail patients. The p-value for sex indicates a comparison between men and women. Values with statistically significant differences ($p < 0.05$) are highlighted in bold (Bonferroni-adjusted).

Supplementary Table S2. Detailed comparison between clinical diagnosis upon admission and segmental phase angles.

Phase angle	Diagnosis				
	Arrhythmias n=16	Infective endocarditis n=2	Heart failure n=34	Coronary artery disease n=96	Valvopathies n=9
	$\overline{X}$ (SD)	$\overline{X}$ (SD)	$\overline{X}$ (SD)	$\overline{X}$ (SD)	$\overline{X}$ (SD)
PHASE°LBD	**5.13** * (0.82)	7.10 (2.55)	**4.81** ** (0.83)	**5.32*** (0.73)	**4.21***** (0.78)
PHASE°RRG	**4.30**** (1,10)	4.00 (0.57)	**4.13**** (1.23)	4.88 (0.97)	**3.78*** (0.60)
PHASE°LLG	4.38 (1.19)	4.00 (0.14)	**4.11**** (1.20)	4.80 (0.90)	**3.62**** (0.65)
PHASE°RAM	**5.54***** (0.84)	8.10 (2.97)	**5.34***** (0.69)	**5.88**** (0.71)	**5.17***** (0.91)
PHASE°LAM	5.63 (0.98)	6.25 (0.07)	5.34 (0.91)	5.71 (0.71)	**4.74**** (0.80)
PHASE°WLG	4.44 (1,10)	4.30 (0.00)	**4.20**** (1.19)	4.93 (0.90)	**3.76**** (0.62)
PHASE°RBD	**4.99***** (0.79)	8.30 (4.10)	**4.82***** (0.81)	**5.40***** (0.73)	**4.49**** (0.77)

Note: $\overline{X}$: mean; SD: standard deviation; PHASE ° LBD: phase angle of the left half of the body; PHASE ° RRG: phase angle of the right leg; PHASE ° LLG: phase angle of the left leg; PHASE ° RAM: phase angle of the right arm; PHASE ° LAM: phase angle of the left arm; PHASE ° WLG: phase angle of both legs; PHASE ° RBD: phase angle of the right half of the body. * p <0.05; **p <0.01; ***p <0.001 (Bonferroni-adjusted).

Supplementary Table S3. Detailed comparison between frailty status, sex, and segmental phase angles.

Segmental phase angles	$\overline{X}$ (SD)	Frailty status (Fried) Frail (a) $\overline{X}$ (SD) p-value	Non-frail (b) $\overline{X}$ (SD) p-value	Pre-frail (c) $\overline{X}$ (SD) p-value	Sex	Frail (a) $\overline{X}$ (SD) p-value	Non-frail (b) $\overline{X}$ (SD) p-value	Pre-frail (c) $\overline{X}$ (SD) p-value
PHASE°LBD	5.15 (0.87)	**4.82** (0.98) c (0.009)	5.23 (0.74)	5.30 (0.80)	Male	**5.00** (1.06) c (0.045)	5.34 (0.76)	5.51 (0.82)
					Female	4.49 (0.75)	4.80 (0.43)	4.95 (0.65)
PHASE°RRG	4.58 (1.09)	**4.13** (1.05) c (0.002)	4.65 (0.76)	4.81 (1.14)	Male	**4.09** (1.08) c (0.018)	4.73 (0.77)	4.82 (1.25)
					Female	4.20 (1.03)	4.32 (0.65)	4.79 (0.96)
PHASE°LLG	4.53 (1.04)	**4.05** (1.00) c (0.000)	4.56 (0.84)	4.78 (1.06)	Male	**4.03** (0.90) c (0.004)	4.64 (0.88)	4.83 (1.15)
					Female	4.09 (1.18)	4.23 (0.58)	4.69 (0.91)
PHASE°RAM	5.71 (0.84)	**5.46** (1.12) c (0.044)	5.77 (0.59)	5.84 (0.72)	Male	**5.63** (1.25) c (0.004)	5.80 (0.61)	6.07 (0.65)
					Female	5.14 (0.77)	5.62 (0.50)	5.46 (0.66)
PHASE°LAM	5.57 (0.82)	**5.21** (0.76) b (0.011) c (0.003)	5.76 (0.80)	5.70 (0.81)	Male	5.38 (0.75)	5.87 (0.82)	5.98 (0.80)
					Female	4.91 (0.69)	5.32 (0.53)	5.23 (0.59)
PHASE°WLG	4.65 (1.03)	**4.20** (1.00) c (0.001)	4.77 (0.76)	4.89 (1.07)	Male	**4.17** (0.96) c (0.008)	4.74 (0.79)	4.91 (1.16)
					Female	4.24 (1,11)	4.37 (0.60)	4.85 (0.91)
PHASE°RBD	5.22 (0.94)	4.96 (1.27)	5.29 (0.63)	5.34 (0.78)	Male	5.17 (1.46)	5.37 (0.66)	5.49 (0.81)
					Female	**4.56** (0.71) c (0.045)	4.98 (0.44)	5.08 (0.67)

Note: $\overline{X}$: mean; SD: standard deviation; PHASE ° LBD: phase angle of the left half of the body; PHASE ° RRG: phase angle of the right leg; PHASE ° LLG: phase angle of the left leg; PHASE ° RAM: phase angle of the right arm; PHASE ° LAM: phase angle of the left arm; PHASE ° WLG: phase angle of both legs; PHASE ° RBD: phase angle of the right half of the body. Significance level for groups (a, b, and c): $p < 0.05$. For each pair of significance, the largest proportion of the frailty status group is indicated. Statistically significant values for $p < 0.05$ are presented in bold (Bonferroni-adjusted).

Supplementary Figure S1. A comparison of ROC curves of segmental phase angles in the overall sample versus frailty status.

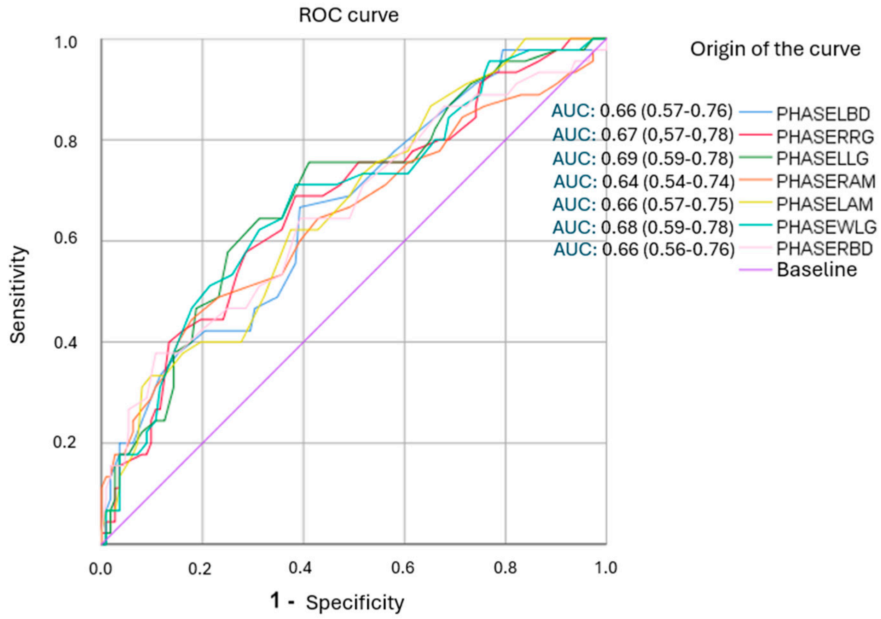

Note: PHASE° LBD: angle of the left half of the body; PHASE° RRG: angle of the right leg; PHASE° LLG: phase angle of the left leg; PHASE° RAM: phase angle of the right arm; PHASE° LAM: phase angle of the left arm; PHASE° WLG: phase angle of both legs; PHASE° RBD: phase angle of the right half of the body.

Supplementary Figure S2. A comparison of ROC curves of segmental phase angles in the overall sample versus frailty status in men.

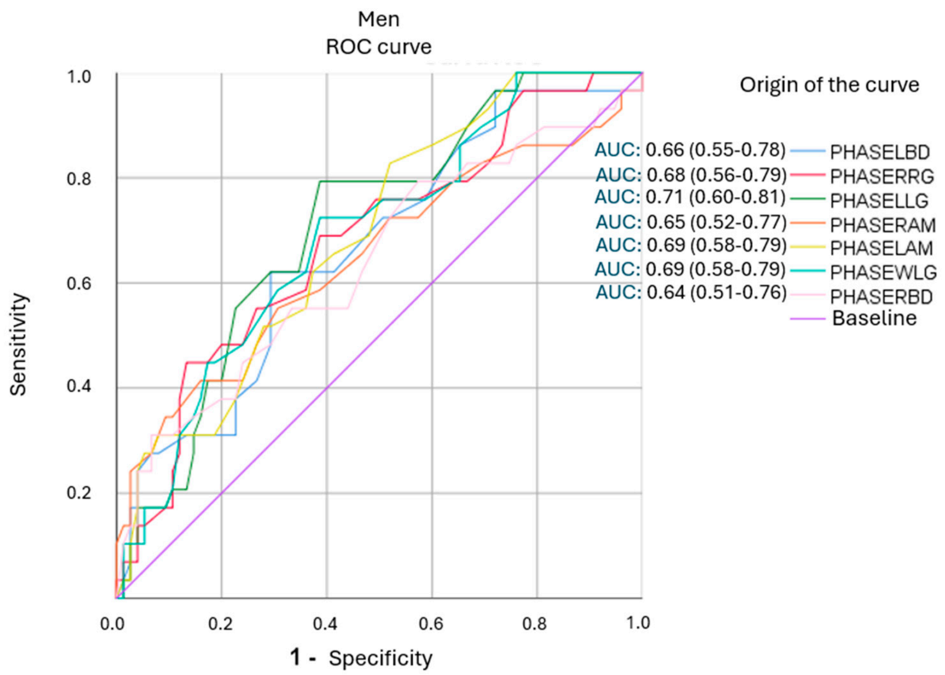

Note: PHASE° LBD: phase angle of the left half of the body; PHASE° RRG: phase angle of the right leg; PHASE° LLG: phase angle of the left leg; PHASE° RAM: phase angle of the right arm; PHASE° LAM: phase angle of the left arm; PHASE° WLG: phase angle of both legs; PHASE° RBD: phase angle of the right half of the body.

Supplementary Figure S3. A comparison of ROC curves of segmental phase angles in the overall sample versus frailty status in women.

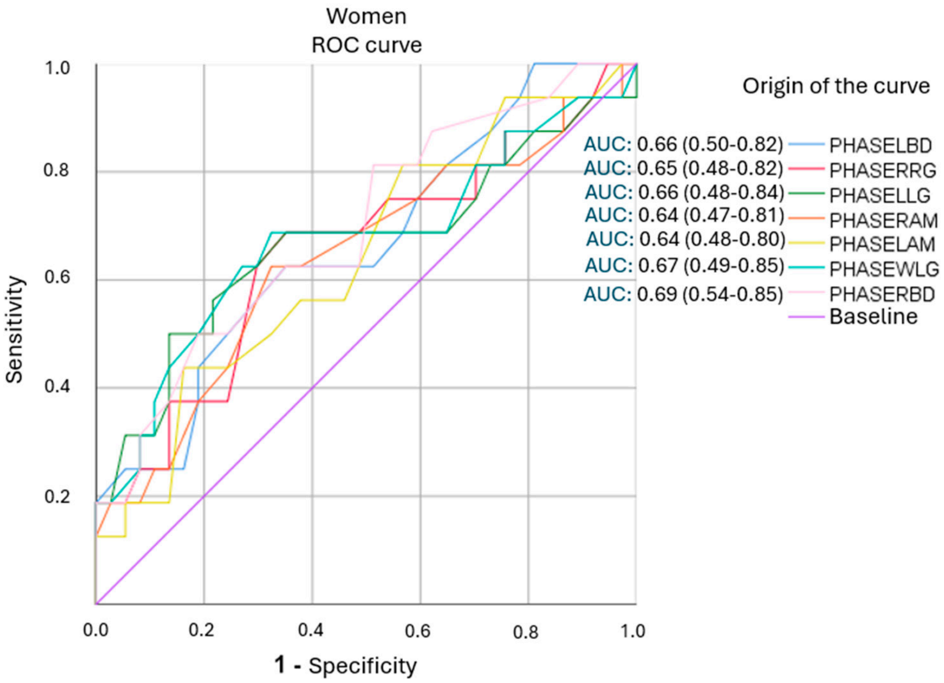

Note: PHASE° LBD: phase angle of the left half of the body; PHASE° RRG: phase angle of the right leg; PHASE° LLG: phase angle of the left leg; PHASE° RAM: phase angle of the right arm; PHASE° LAM: phase angle of the left arm; PHASE° WLG: phase angle of both legs; PHASE° RBD: phase angle of the right half of the body.

Supplementary Discussion S1

Regarding body composition, the mean body mass index of the sample was within the overweight range, with a higher percentage in frail patients in both sexes. However, when analyzing the sarcopenia index adjusted by sex, values were within the normal range in all groups, and slightly higher in patients with frailty, according to the cut-off points proposed by the European Group and the International Sarcopenia Group [37,38]. This finding suggests that the loss of muscle mass was not a determining factor in the state of frailty in this hospital cohort, aligning with studies that have described a disconnect between mass and function in acute clinical settings [11]. Additionally, abdominal perimeter was higher in men with frailty and pre-frailty, consistent with the findings of Crow et al. [46], who identified a link between central obesity and frailty in older adults. These results indicate a frailty profile more closely associated with excess adiposity than with loss of lean mass, particularly in men. This reinforces the importance of evaluating body fat distribution as part of the comprehensive assessment of frail patients with CVD.

However, it should be highlighted that although sarcopenia was not prevalent in this sample, its role in patients with CVD is well documented. The absence of clinically relevant sarcopenia in this cohort may reflect the acute clinical context and selection of patients capable of standing for BIA assessment. Several studies have indicated that decreased muscle mass and strength are associated with an increased risk of adverse cardiovascular events and mortality, even in the presence of an apparently normal BMI [3,13]. In patients with heart failure, sarcopenia contributes to functional impairment and exercise intolerance and may coexist with obesity in what has been termed sarcopenic obesity, a condition with a worse prognosis. In this sense, the absence of sarcopenia in the present cohort could reflect a specific clinical selection, the stage of the disease, or even limitations of the bioimpedance-based measurement method, as recently discussed [20]. This finding also suggests that EWGSOP2 and IWGS criteria may have limited applicability in acutely hospitalized patients, where temporary fluid or mobility alterations can mark muscle decline. Therefore, although our data do not show a direct impact of sarcopenia on frailty at admission, its systematic evaluation remains a priority in the care of older patients with CVD.

Regarding body fat, we observed that patients with frailty had a higher percentage of total and segmental fat, especially in the right arm, where statistically significant differences were found. Although the average percentage of body fat in men was below the threshold proposed by Tanaka et al. as a reference value for the development of frailty [18], in women, it exceeded this value (32.15%) without reaching statistical significance. The presence of sarcopenic obesity was low in both sexes, being identified in only 6.63% of men, with no cases in women. This pattern suggests that,

in the analyzed sample, fat accumulation may have played a more relevant role than muscle mass loss as a component of frailty, especially in men. However, these results should be interpreted with caution, as several studies have highlighted the role of decreased lean mass in the progression to frailty. In this cohort of hospitalized older adults with CVD, the only significant association was observed with the percentage of segmental fat in the right arm, suggesting a more relevant fat–muscle distribution pattern than the overall loss of lean mass [11].

The literature supports the role of sarcopenic obesity as a metabolically adverse condition, associated with chronic inflammation, insulin resistance, and increased cardiovascular risk, making it a high-risk clinical phenotype in older adults with CVD [3,39]. Furthermore, it has been noted that body composition in women may be more influenced by hormonal factors and differences in subcutaneous fat distribution, which could explain the lower diagnostic sensitivity of phase angles in this group. The low prevalence of sarcopenic obesity in this cohort could be related to the hospital setting, baseline functionality, or inclusion bias, but its potential clinical impact should not be underestimated, especially in more advanced stages of cardiovascular disease.
